# A Medical Programme

**Published:** 1894-01-13

**Authors:** 


// The Hospital

An Institutional Journal of

The Medical Sciences and Hospital Administration, with

Vol. XV.—No. 381.] AN EXTRA NURSING SUPPLEMENT. [Jan. 13, 1894.

### A Medical Programme.

The medical journalist of the *fin de siècle* finds himself in the midst of a strenuous time and of eager battalions of strenuous workers. Not the medical writer only, but the practitioner, whether in town or country, feels the pressure of new ideas, and sometimes a sense of paralysing confusion from the infinite diversity of modern opinion. But in the profoundest intellectual chaos there is order to be found by the intellect which is capable of finding it. The daily practice of medicine imperatively demands that order be found. Nowhere is indecision more baneful than at the bedside. The practising doctor must be, above all things, a man of knowledge and of authority.

It is a high responsibility to teach the teacher, to lead the guide. That responsibility has gradually devolved upon the conductors and writers of The Hospital. We need not go back, as we did at the beginning of last year, upon our past history. The facts are to be accepted as they stand. It has been our endeavour to open the hospital ward to the private practitioner; and this opening of the hospital ward has introduced an element of order and certainty into the often confused and confusing medical opinion of the time. Once arrived at the bedside of the patient in the hospital, the consciousness asserts itself that the time for speculation is passed for the moment, and that the time for action has arrived. The hospital physician or surgeon feels that though he may not be able always to apply in present practice the profoundest scientific principles he has theoretically mastered, he must, in loyal earnestness apply those which a previous science and a competent experience have approved. He ceases, therefore, for the time to be a speculative theorist, and becomes a man of resolute decision and action. It has been our aim for many months past to open the ward doors upon such workers as these, in order that those who are struggling single-handed with the responsibilities of private practice may have object lessons of the most practical and helpful kind constantly brought before them from week to week.

But practice without knowledge is little better than a beating of the air, and in the case of medicine the beating of the air is not only unprofitable but dangerous. Never were there such facilities for obtaining medical knowledge as there are at the present time. It is almost impossible, even for the least cultured of practitioners, to be absolutely without knowledge. But because knowledge is so abundant, and almost superabundant, there is all the more reason why, for the busy practitioner, it should be selected, sifted, and duly labelled, in order that the few precious hours he can spare in the week for reading may not be wasted. The only result to many a man, of wading through confused masses of undigested thought and theory, is that in his overworked brain confusion becomes more and more hopelessly confounded. That is a result to be averted and avoided at all costs. As we have already stated, the medical man must above all things be a man clear in ideas and resolute in action.

It has been the good fortune of The Hospital to awaken the sympathy of many members of the medical profession, of whom it may be said that their distinguishing characteristic is clearheadedness. They have the power of differentiating between the essential and the accidental, and also between the less essential and the more essential. Several of those eminent members of the profession have undertaken to write systematically and simply on several subjects of immediate practical interest. They have been sifting and selecting for their own personal use for many years; and the definite results of their selective processes, labelled with the stamp of their experience and approval, they will set before our medical readers in brief and pointed form from week to week. It is the intention of these writers to be of practical service to their brethren. Hating crudity and confusion in their own work, they sympathise earnestly with those who, in the hurry and pressure of practical business are so liable to become the victims of literary inexperience and crudity. With writers of clear intellect and experience to set before readers the facts and principles, old as well as new, upon which modern medical art and science are based, and an interesting presentation of every variety of special and genera ward work from week to week, we hope to furnish forth a continuous programme of so concentrated and useful a character as medical readers have not hitherto been able to find elsewhere.

It is not our desire to make a boast of special sympathy with any one class of practitioners. But we feel that no man who has read deeply in medical literature, and who comprehends, even in a limited degree, the profound scientific problems and the serious practical difficulties which beset modern medicine on all sides, can fail of wide sympathy, or of a desire to push forward practical progress on the widest possible scale. In all professions competition makes life an arduous and weary struggle. In none is the struggle more keenly felt than in medicine. The modern medical man must be modern, or he will fail to survive in the struggle. It is our wish to help him to be modern: modern in the best sense; to arm him with all the experience of the past, and all the science and learning of the present. With competent writers and sympathetic readers this can be done.

Specialism is the order of the day. The family practitioner and the general physician must each add a knowledge of the various specialisms to his peculiar resources. No man, of course, can be an universal specialist; but every man can give his own patients the advantages of universal specialism, by knowing exactly when and wherefore a specialist is needed. One thing more he must know, namely, what specialists are worthy of his own and his patients' confidence. For it has happened with specialisms, as with all institutions for which there is a wide public demand, that the "precious" has got largely mixed with the "vile," and great knowledge and discrimination are required for their separation. The patient himself never possesses this knowledge and discrimination; but every practising doctor ought to possess it. It is our desire to furnish him with the means of acquiring and maintaining it. A certain class of so-called specialists are ripe for suppression. It is for the general physician and surgeon, whether of the consulting or family variety, to sternly suppress them. On the other hand, there are classes of specialists who are absolutely indispensable both to the scientific progress of medicine, and to the perfect practice of the art. These it is the general physician's duty to support and encourage by all legitimate means. We hope that in this useful service also we shall always be found able and willing to render material help.

The modern hospital has gradually become the head and front of modern medicine. It is indispensable, therefore, for every practitioner, whose purpose it is to be modern and abreast of the times, to maintain a constant intercourse and familiarity with the works and ways of hospitals. There are only two methods by which this can be accomplished. The one is by personal attendance in the wards; the other is by reading and studying diligently from day to day and from week by week the adequately described work of the wards. Where can any descriptions be found which are at once so full and so concisely brief as those given in our own pages? We are The Hospital. The hospital, not in material form, but in spirit and essence. Our chief, our only *raison d'être*, is the presentation of the actual hospitals of the world, in all their living and varied activity, to that great and increasing body of workers whose business is the care and cure of the sick. That this bringing of the hospital wards and their work into the consulting-room of the busy practitioner is a work worthy and imperatively necessary to be done, we are well assured; that we have hitherto accomplished it in such a way as entirely to satisfy our own ideals we dare not affirm; but that so far as capacity, opportunity, and determination may avail, we shall better realize our ideals in the time to come, we trust that neither ourselves nor our sympathetic readers have any manner of doubt.

